# Disentangling the Multifactorial Influences on Diabetes Risk Among Rural Communities: Food Environment, Diet Quality, and Dietary Chemical Exposures

**DOI:** 10.1002/dmrr.70177

**Published:** 2026-05-09

**Authors:** David C. Lee, Haley L. Motola, Jessie Moore, Tammy Flores, Crystal So, Haeseung Yi, Vittorio Albergamo, Leonardo Transande, Brian Elbel, Lorna E. Thorpe

**Affiliations:** ^1^ Department of Emergency Medicine NYU School of Medicine New York New York USA; ^2^ Department of Population Health NYU School of Medicine New York New York USA; ^3^ Sullivan County Public Health Services Liberty New York USA; ^4^ Department of Pediatrics NYU School of Medicine New York New York USA

**Keywords:** bisphenols, chemical hazards, diabetes risk, phthalates, rural health

## Abstract

**Aims:**

Rural communities experience a higher prevalence of type 2 diabetes and diabetes‐related mortality than urban populations. This study sought to disaggregate the influences of demographic and socioeconomic factors, food environment, diet quality, and dietary chemical exposures on diabetes risk in rural areas.

**Materials and Methods:**

We enrolled participants from rural Sullivan County in an observational cohort study involving surveys and biospecimen collection measuring bisphenols and phthalates. We measured these endocrine disrupting chemicals found in food packaging, as rural residents generally consume canned foods and other shelf‐stable foods more frequently than their urban counterparts. We used LASSO regression to compare the relative influence of these factors had on rural diabetes risk.

**Results:**

Based on values for LASSO regression coefficients among 276 participants, the strongest risk factors for diagnosed diabetes included: older age (+0.486), lower household income (+0.172), Hispanic ethnicity (+0.124), red meat intake (+0.093), proportion of fast food restaurants among nearby restaurants (+0.071), and two phthalates (+0.149 and + 0.107). Among study participants without a history of diabetes, high HbA1c levels were associated with older age (+0.106), being non‐Hispanic Black (+0.064), more trans‐fat and red meat intake (+0.044 and +0.028), higher BMI (+0.014), higher levels of total bisphenols (+0.005), and higher levels of high‐molecular weight phthalates (+0.002).

**Conclusions:**

Demographic and socioeconomic factors were the strongest predictors of rural diabetes risk; however, diet quality, food environment, and dietary chemical exposures also each played a key role. Our study identified modifiable risk factors, which could help reduce the burden of rural diabetes.

## 1 Introduction

Over the past 2 decades, diabetes prevalence has increased across the United States [1]. However, mortality due to diabetes has been persistently higher in rural areas, with a 1.7 times higher rate of diabetes‐related deaths, as compared to urban areas [2, 3]. Many factors are thought to contribute to these poor rural health outcomes, including unhealthy food environments, poor diet quality, less frequent physical activity, and barriers to healthcare access. However, dietary hazards not only include an unhealthy imbalance of macronutrients, such as high carbohydrates and fats, but also dietary contaminants, such as bisphenols and phthalates, present in certain foods [4, 5]. These chemicals are recognised as endocrine disruptors that promote insulin resistance [5, 6].

Rural communities face an especially high burden of diabetes and severe economic and geographic barriers in accessing healthy foods. While crude measures of unhealthy food environments are able to capture increased diabetes risk in urban areas, they are not always directly applicable to rural areas [7, 8, 9]. Robust studies of the food environment and rural diabetes risk are needed to measure exposures at the correct geographic scale and quantify how certain food purchases can increase diabetes risk for especially high‐risk populations [8, 9]. As for dietary chemical exposures, rural residents are known to consume more canned and processed foods than urban residents due to their longer shelf life and lower cost [10, 11, 12]. When compared to foods in other types of packaging, canned foods contain levels of bisphenols that are 15 times higher due to their plastic lining [13, 14]. While there have been efforts to label and reduce bisphenol A (BPA), the use of other synthetic bisphenols to replace BPA is on the rise [15]. Therefore, a given food purchase can be hazardous not only due to its dietary content, but also from chemicals hazards such as bisphenol and phthalates that promote serious diseases like diabetes [16, 17]. Though the effects of poor diet quality and dietary contaminants have been studied separately, few studies have examined the relative contribution of both hazards on diabetes risk among rural residents.

The goal of this study was to assess how the built food environment, poor diet quality, and dietary contaminants affect diabetes risk in rural communities. We hypothesised that each would have a statistically significant influence on rural diabetes risk even after adjusting for socioeconomic factors that drive most of the poor health outcomes in rural regions of the United States [18, 19].

## 2 Materials and Methods

### 2.1 Study Design

This study was the third and final phase of a multi‐part NIH funded study, which assessed diabetes risk in a rural region of New York State. In the first stage, a brief health survey was delivered by mail to all 28,284 households in Sullivan County. This was followed by a second stage in which 3392 of 5230 initial brief health survey respondents (the rest stating on the initial survey that they did not want to participate further) were invited to provide more detailed socioeconomic, health and dietary data through a longer survey and a food frequency questionnaire (FFQ) [20]. Finally, 304 of 1446 second stage survey respondents attended the third stage with an in‐person visit for biospecimen collection, where they provided blood and the first of four urine samples. (A complete flow diagram for participation in each of these three stages is available at a related publication cited here [21]). The remaining three urine samples were self‐collected at home and mailed back to the study team for processing and analysis. Participants also repeated previously completed surveys and FFQs to assess the consistency of their responses at the time of biospecimen collection.

### 2.2 Study Setting and Participants

The study was conducted in Sullivan County, New York, which was ranked at the time as having the second worst health outcomes in the entire state [22]. We focused on a single county in order to perform a geographically detailed study using food environment metrics that captured exposures at the correct geographic scale. This approach allowed us to study how the food environment varied on a within‐county basis among this cohort of rural study participants [23]. The results of the first two stages of this study have confirmed the high‐risk nature of the study participants as we found very high rates of food insecurity and other adverse socioeconomic conditions within this rural county [24].

### 2.3 Primary Outcome

The primary outcome of our study was to identify risk factors for rural diabetes, which we assessed in two different ways. First, in line with prior studies that have identified dietary chemical exposures (e.g., bisphenols and phthalates) as endocrine disruptors, we used a self‐reported diagnosis of type 2 diabetes as one of our primary outcomes [25, 26, 27]. In these analyses, we excluded individuals with a known diagnosis of type 1 diabetes. Second, we also assessed the diabetes risk by evaluating glycaemic control as measured by HbA1c levels collected among the study participants who did not have a known diagnosis of diabetes.

### 2.4 Predictor Variables

As for variables that predicted the diabetes risk among our rural study population, we included the following variables in each of our analyses: demographic and socioeconomic variables including age, sex (male or female), race/ethnicity (White, Black, Hispanic, or Other), and household income (in categories of less than $24,999, $25,000 to $49,999, $50,000 to $99,999, and $100,000 or more). In addition, we asked each study participant whether they were physically active with the question: “Are you physically active? Approximately 150 min of moderate intensity or 75 min of vigorous intensity exercise each week.” Height and weight measurements were also obtained to calculate body‐mass‐index (BMI).

The food environment was analysed among k‐nearest neighbours, an approach that accounted for geographic scale. For each study participant, we categorised the nearest 20, 10, and 5 restaurants as either fast food or not and included the proportion of fast food restaurants as a predictor of diabetes risk. Similarly, we categorised the nearest 20, 10 and 5 retail food stores as grocery/supermarkets, dollar stores, and convenience stores and included the proportion of grocery/supermarkets as a predictor of diabetes risk. These choices were made based on analyses from the second phase of our study, where we found nearest neighbour metrics to be optimal measures of the rural food environment [28].

As for diet quality, we used the data from the FFQs to calculate alternative Healthy Eating Index (aHEI) 2010 scores by first determining the intake of the 11 subcomponents of the aHEI 2010 (e.g., vegetables, sugar‐sweetened beverages, alcohol). We assigned a subcomponent score for each of the categories based on intake level and summed the component scores to calculate a total score, which can range from 0 to 110 with a higher score indicating greater adherence to a healthy dietary pattern. Both the total aHEI 2010 scores and the 11 subscores were included in our analyses as predictors of diabetes risk.

As for dietary chemical exposures, we tested pooled samples for 4 days of urine biospecimen collected both in‐person and sent by study participants. Mailed urine samples were self‐collected for an additional 3 consecutive days given the short‐half lives of some of the chemicals tested. We used liquid chromatography coupled with mass spectrometry to test a panel of eight bisphenols and 23 phthalates/phthalate metabolites, three non‐phthalate replacement metabolites (related to DINCH) and phthalic acid. All samples were analysed by the Hazards Human & Environmental Exposure Assessment Lab at NYU. We excluded five bisphenols and four phthalates as predictors given that they were not detectable in over 99% of the participants studied. We also included the sum of all bisphenols, the sum of all low‐molecular weight phthalates, the sum of all high‐molecular weight phthalates, and the sum of food packaging related phthalates as additional predictors in line with other research studies (see Supporting Information S1: Table for the list of all chemicals analysed). For those participants with undetectable chemical levels, we used the limit of detection (LOD) divided by the square root of 2 as a replacement value.

### 2.5 Statistical Analysis

We first described the study population by age, sex, race/ethnicity, income, aHEI scores, food environment, and HbA1c levels. We also summarised the range of bisphenol and phthalate levels using median, interquartile ranges, and 5th and 95th percentile values. To provide additional context for these chemical hazard levels, we also compared the 50th, 75th, 90th, and 95th percentile values for several chemicals tested in our rural study population that were also tracked by the National Health and Nutrition Examination Survey (NHANES) for adults aged 20 years and older.

To identify risk factors associated with a higher prevalence of type 2 diabetes and high HbA1c levels among those without a known history of diabetes in our rural study population, we used Least Absolute Shrinkage and Selection Operator (LASSO) regression given the large number of independent variables tested and to provide a comparative understanding of the relative contributions of each factor. A logit function was used for diagnosis of type 2 diabetes, and a linear function was used for HbA1c level among those without a known diagnosis of diabetes. Demographic and socioeconomic predictors included age (as a continuous variable), sex, race/ethnicity, and household income. The food environment was assessed based on the proportion of restaurants that served fast food and also the proportion of retail food stores that served grocery stores or supermarkets [28]. Given the skewed distribution of the chemical hazards, we log transformed the values using base 2 to provide the predictors as doubling of levels. In addition, we controlled for self‐reported physical activity as a binary variable and body‐mass‐index as a continuous variable. Statistically significant predictors were identified based on the magnitude of the LASSO regression coefficient, which is an indicator of the strength of the variable in predicting the outcome.

All statistical analyses were performed using STATA 19.5 (StataCorp: College Station, TX). The Institutional Review Board at the NYU School of Medicine approved this study under the study protocol s19‐01920. All study participants provided signed informed consent prior to being involved in the research.

## 3 Results

### 3.1 Study Participants

We enrolled a total of 304 study participants in this third and final phase of our study of rural diabetes risk. In our analyses, we excluded 25 participants who did not complete the FFQ and one participant who did not complete the health surveys. Of the remaining 278 study participants, two had a known diagnosis of type 1 diabetes, 31 had a known diagnosis of type 2 diabetes, and 6 reported a known diagnosis of diabetes but did not know which type. In all analyses, the individuals with type 1 diabetes were excluded for a final analytic sample size of 276, and those reporting unknown type were assumed to have type 2 diabetes.

Comparing study participants without a history of diabetes to those with diagnosed type 2 diabetes, we found that participants with diabetes were older (median age 67 vs. 61), non‐White, and in lower income brackets compared to those study participants without diabetes (Table 1). Diet quality, as assessed by total aHEI scores, was similar between those with and without diabetes. As for the food environment, participants with diabetes were living in neighbourhoods with a higher proportion of fast food restaurants but also a higher proportion of grocery stores or supermarkets compared to those participants without diabetes. As expected, HbA1c levels were higher among those with diabetes compared to those without a known diagnosis of diabetes (median HbA1c of 6.0% vs. 5.4%), but within each group, the range of HbA1c levels was relatively wide (4.2%–7.0% in the overall study population).

**TABLE 1:** Study population characteristics.

Population characteristics	Study participants analysed	Diagnosed diabetes (excluding type 1)	No prior history of diabetes
Total number	276	38	238
Age			
Median	61	67	61
Range	28 to 93	37 to 93	28 to 85
Sex			
Male	33%	34%	32%
Female	67%	66%	68%
Race/Ethnicity			
NH White	86%	68%	89%
NH Black	3%	8%	3%
Hispanic	9%	21%	6%
Other	2%	3%	2%
Income			
Less than $24,999	20%	32%	17%
$25,000 to $49,999	22%	42%	19%
$50,000 to $99,999	29%	10%	32%
More than $100,000	29%	16%	32%
Physically active			
Yes	62%	50%	64%
No	38%	50%	36%
Body‐Mass‐Index			
Median	28.4	30.3	27.8
Range	15.9 to 59.2	21.8 to 43.3	15.9 to 59.2
aHEI scores			
Median	57	56	58
Range	28 to 86	36 to 81	28 to 86
Fast food (20 nn)			
Median	25%	30%	20%
Range	0%–45%	5%–45%	0%–45%
Grocery stores & supermarkets (20 nn)			
Median	30%	35%	30%
Range	10%–65%	15%–50%	10%–65%
HbA1c Levels			
Median	5.4%	6.0%	5.4%
Range	4.2%–7.0%	4.4%–11.1%	4.2%–7.0%

### 3.2 Chemical Hazard Levels

**FIGURE 1:**
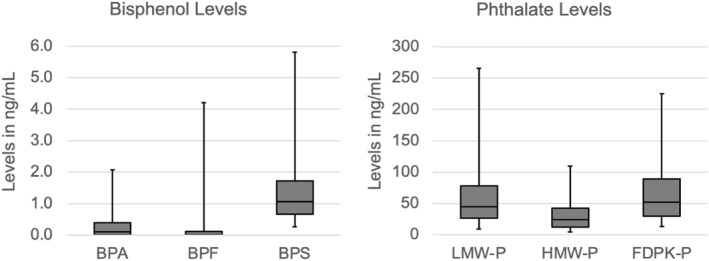
Urinary concentrations of bisphenols and phthalates. Bisphenol A (BPA), bisphenol F (BPF), bisphenol S (BPS), low‐molecular weight phthalates (LMW‐P), high‐molecular weight phthalates (HMW‐P), food packaging phthalates (FDPK‐P).

Bisphenol A, bisphenol F, and bisphenol S were each detected in the study population. Approximately half (51%) of study participants did not have a detectable level of bisphenol A and 76% of study participants did not have a detectable level of bisphenol F. In comparison, all study participants had a level of bisphenol S that was higher than the limit of detection and the range of values for bisphenol S were higher than for bisphenol A or bisphenol F (Figure 1). Given the number of phthalates tested, we summarised the levels for low‐molecular weight phthalates (LMW‐P), high‐molecular weight phthalates (HMW‐P), and food packaging phthalates (FDPK‐P). There was a wider range of values for the sum of low‐molecular weight phthalates compared to high‐molecular weight phthalates.

**FIGURE 2:**
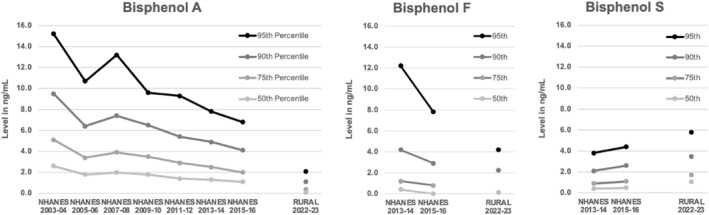
Comparisons of bisphenol levels to historical NHANES data.

The most recent NHANES data is several years older than chemical hazard levels found in our rural study population; by 7 years for bisphenols, and 5 years for phthalates. Furthermore, bisphenol F and bisphenol S have only recently been tracked by NHANES starting in 2013–2014 and there have only been two data points so far. For bisphenol A, levels in our rural study population were in line with historical decreases tracked in the past decade. Bisphenol F in our rural study population was lower than the two historical estimates found in the estimated NHANES data. For bisphenol S, our data suggest an increasing trend relative to prior NHANES estimates (Figure 2). For individual phthalates tested, all tracked phthalates and non‐phthalate alternatives were either lower or stable in our rural study population relative to historical data (see Supporting Information S1: Figures S1–S3).

### 3.3 LASSO Regression Results

In our regression analyses, the strongest predictor of a diagnosis of diabetes (excluding type 1) was older age (LASSO coefficient of +0.485). This was followed by other demographic and socioeconomic factors such as non‐Hispanic White (associated with lower risk at −0.275), income of $25,000‐$49,999 (higher risk at +0.176), income of $50,000–$99,999 (lower risk at −0.143), and Hispanic ethnicity (higher risk at +0.140). However, two specific phthalates were also associated with a higher risk of diabetes in our rural study population: mono‐hexyl phthalate (+0.153) and mono‐2‐ethylhexyl phthalate (+0.115). While overall diet quality and certain subcategories, such as sugar sweetened beverages, did not predict a higher risk of type 2 diabetes, we noted that less red meat (−0.093) was a protective factor, in addition to being more physically active (−0.089). Finally, a higher proportion of fast food restaurants (based on the 20 nearest neighbours) also conferred a higher risk of type 2 diabetes (+0.061) (Table 2).

**TABLE 2:** LASSO regression coefficients.

Diabetes diagnosis (excluding type 1) (*n* = 276)		HbA1c Level if No history of diabetes (*n* = 238)	
Predictors	LASSO Coefficient	Predictors	LASSO Coefficient
Older age	+0.485	Older age	+0.107
Non‐Hispanic White	−0.275	Non‐Hispanic Black	+0.063
Income $25–49K	+0.176	Less trans fats	−0.042
Mono‐hexyl phthalate	+0.153	Less red meat	−0.026
Income $50–99K	−0.143	Non‐Hispanic White	−0.026
Hispanic	+0.140	Body‐Mass‐Index	+0.014
Mono‐2‐ethylhexyl phthalate	+0.115	Total bisphenols	+0.006
Less red meat	−0.093	High‐molecular weight phthalates	+0.002
Physically active	−0.089	Mono‐butyl Phthalate	+0.001
Fast food (20 nn)	+0.061		

In addition, we analysed which predictors were associated with poorer glycaemic control (higher HbA1c level) among rural study participants without a prior diagnosis of diabetes. Of these 238 study participants, 53 (22%) had a HbA1c level consistent with prediabetes (HbA1c of 5.7%–6.4%) and 3 study participants had a HbA1c level consistent with previously undiagnosed diabetes (HbA1c levels of 6.5% or higher). In our regression analysis, we also found that older age was the strongest predictor of poor glycaemic control among those without a known diagnosis of diabetes (LASSO coefficient of +0.107). Race was also a statistically significant predictor, with non‐Hispanic Black race being associated with higher risk (+0.063) and non‐Hispanic White race being associated with lower risk (−0.026). In addition, higher BMI (+0.014) was associated with poor glycaemic control, and dietary factors such as less intake of trans fats (−0.042) and less intake of red meat (−0.026) were both associated with better glycaemic control. Finally, we also found that specific chemical hazards also predicted poorer glycaemic control among rural residents without a known diagnosis of diabetes, although the magnitude of association was not large. These included the total level of bisphenols (+0.006), the sum of high‐molecular weight phthalates (+0.002) and mono‐butyl phthalate (+0.001).

## 4 Discussion

The goal of this study was to assess how the food environment, poor diet quality, and dietary contaminants affect diabetes risk in rural communities. We sought to disaggregate the individual contribution of these factors to understand their relative impact on rural diabetes risk. Most studies either analyse one factor without the others or conclude that the cause of diabetes is multi‐factorial, in other words, difficult to assess [29, 30, 31, 32]. While there are indeed a variety of factors that affect diabetes risk in rural communities, it is critical to understand the relative importance of each influence. While our study found that demographic factors drive much of the diabetes risk in our rural study population, factors like food environment, diet quality, and dietary chemical exposures each play a significant role in rural diabetes risk as well.

We hypothesised that a given food purchase can be hazardous and increase diabetes risk not only due to its dietary content in terms of macronutrients but also from the chemicals that can leech into these foods from packaging or other sources. Currently, many studies have demonstrated the endocrine disrupting potential of bisphenols and phthalates [33, 34, 35]. The strongest evidence of this potential exists for bisphenol A and for metabolites of di‐2‐ethylhexyl phthalate (DEHP), which include mono‐(2‐ethyl‐5‐hydroxyhexyl) phthalate (mEHHP) and mono‐(2‐ethyl‐5‐oxohexyl) phthalate (MEOHP) among others [29, 30, 36]. Given this evidence, bisphenol A and many phthalates have been largely phased out or replaced. Even in the rural population studied here, levels for chemical hazards were much lower than historical trends from NHANES data and more than half of our study population had no measurable level of bisphenol A.

However, our study also provides preliminary evidence that the use of bisphenol replacements, such as bisphenol S, may be on the rise. In our study, we found evidence that the sum total of bisphenols was associated with poorer glycaemic control among those rural study participants without a known diagnosis of diabetes. With declining levels of bisphenol A, bisphenol S on average accounted for over 60% of the total bisphenol levels for our rural study participants. While the metabolic effects of bisphenol A have been extensively studied, the research on bisphenol S has been limited, and it may prove to be just as endocrine disrupting or potentially more so [37, 38, 39]. Indeed, once national surveillance studies begin to track chemical hazards, chemical companies move quickly to replace them with new chemicals, the effects of which are largely unknown. This underscores the need to improve the surveillance of chemical hazards using new technologies and identify the endocrine disrupting potential of replacement chemicals much earlier on.

This study was the final part of a multi‐phase study on rural diabetes risk and was focused on understanding the impact of dietary chemical hazards relative to other risk factors for rural diabetes [40]. Our analysis also confirms many other findings from the other parts of this NIH funded study of rural diabetes risk. For instance, in our prior studies, we found that cases of diagnosed diabetes were geographically clustered in areas with a high density of fast food restaurants but also grocery and supermarkets [20]. In this current study, we also found that the proportion of fast food restaurants predicted the risk of type 2 diabetes, and the proportion of grocery stores and supermarkets was not a predictive factor. This finding runs contrary to the traditional narrative that rural diabetes risk is driven by a lack of access to fresh foods and long distances to retail food stores. Although these barriers to food access may affect certain rural residents, we found that cases of diagnosed diabetes actually cluster in areas near grocery stores and supermarkets [20].

In addition, in one of our prior studies of dietary patterns among rural residents with and without diabetes, we found evidence demonstrated that those with a known history of diabetes were more likely to consume higher amounts of red meat and trans‐fats as well as suboptimal quantities of alcohol and nuts/legumes [41]. In that prior study, we also found a non‐statistically significant trend that those with diabetes consumed less sugar‐sweetened beverages. In the present study, we see confirmatory evidence of these findings, where a lower intake of red meat was associated with a lower risk of type 2 diabetes, and a lower intake of red meat and trans fats were associated with better glycaemic control among those rural residents without a known diagnosis of diabetes. Collectively, these findings suggest that certain dietary choices, such as red meat and trans fats, may be perceived by rural residents as less obvious risk factors for diabetes compared to more familiar risk categories like sugar sweetened beverages or sugary foods. These other dietary risk factors may need more emphasis in order to reduce the burden of diabetes among rural communities.

While the cause of diabetes in rural communities is multi‐factorial, our study disaggregated risks to understand the relative contribution of different risk factors of rural diabetes. While we found that demographic and socioeconomic factors were the strongest predictors of diabetes risk among our rural study participants, the food environment, diet quality, and dietary chemical exposures each independently conferred an increased risk of diabetes. Our study identifies modifiable risk factors, which could help reduce the burden of rural diabetes, especially given the disproportionately high rates of diabetes‐related deaths among rural residents.

### 4.1 Limitations

Our study focused on a single rural county to perform a geographically precise study of the relationship of the food environment, diet quality, and dietary chemical hazards on diabetes risk in rural areas. While the approach allowed us to develop metrics with geographic precision, the findings of our study may not be generalisable to other rural areas of the country. In addition, much of our data was generated based on self‐reported data such as a diagnosis of diabetes, physical activity, household income, and FFQ responses used to calculate diet quality scores, and our analysis is therefore, in part, subject to the limitations of self‐reported data. Our sample size was not as large as national studies on the effect of chemical hazards on diabetes risk; therefore, the absence of any association should not be considered evidence that these chemical hazards or any other measured factors do not pose an elevated risk. Furthermore, the overall study design that was implemented in three stages with an initial brief health survey, a more detailed second survey, and in‐person participation. There may be substantial selection bias such that our final analytic sample in this third stage that makes it not representative of the general population in Sullivan County. Therefore, the results should be considered in light of this limitation of the overall study design. Finally, our study is observational, and any statistically significant associations identified should not be taken as evidence of causation as they may be cofounded by other factors not measured in our study.

## Author Contributions

D.C.L. researched data, contributed to discussion, and wrote the first draft of the manuscript. H.L.M., J.M., T.F., C.S., H.Y., L.T., B.E., and L.E.T. reviewed and edited the manuscript. V.A. researched data and reviewed and edited the manuscript. All authors approved the final version of the manuscript. D.C.L. is the guarantor of this work and, as such, had full access to all the data in the study and takes responsibility for the integrity of the data and the accuracy of the data analysis.

## Funding

This study was funded by grant R01‐DK124400 from the National Institutes of Diabetes and Digestive and Kidney Diseases, which is focused on understanding the geographic and environmental risk factors for diabetes in rural areas of the country.

## Conflicts of Interest

The authors declare no conflicts of interest.

## Supporting information


Supporting Information S1

## Data Availability

The data that support the findings of this study are available from the corresponding author upon reasonable request.
