# Supplementary material for: Disentangling the Multifactorial Influences on Diabetes Risk Among Rural Communities: Food Environment, Diet Quality, and Dietary Chemical Exposures
Source: Diabetes Metab Res Rev. 2026 May 9;42:e70177. doi: 10.1002/dmrr.70177 (PMC13157511; doi:10.1002/dmrr.70177)

**SUPPLEMENTAL TABLE**

| **Chemical Grouping** | **Parent Compounds** | **Metabolites Analyzed**  (Proportion Detectable) | **Excluded but Tested**  (Proportion Detectable) |
| --- | --- | --- | --- |
| Low-Molecular Weight Phthalates  (LMW-P) | DMP, DEP, DiBP, DBP, DnHP, DHPP | mMP (51.3%)  mEP (100%)  mIBP (97.0%)  mBP (100.0%)  mHxP (33.0%) | mHpP (0.3%) |
| High-Molecular Weight Phthalates  (HMW-P) | DEHP, DiNP, DiDP, DnOP, BzBP, DCHP, DPHP | mECPP (99.7%)  mCMHP (68.4%)  mEHHP (95.7%)  mEOHP (95.7%)  mEHP (58.9%)  mCIOP (96.7%)  mCINP (60.5%)  mCPP (34.9%)  mCHpP (24.3%)  mBzBP (62.2%)  mCHP (6.6%)  OH-mPHP (88.8%)  oxo-mPHP (96.7%)  cx-mPHxP (8.9%) | mINP (0%)  mIDP (0%)  mOP (0%) |
| Food Packaging Phthalates  (FDPK-P) | DEP, DEHP, DiNP, DiDP, DCHP, BPBG, DIOP, EPEG | mEP (100%)  mECPP (99.7%)  mCMHP (68.4%)  mEHHP (95.7%)  mEOHP (95.7%)  mEHP (58.9%)  mCIOP (96.7%)  mCINP (60.5%)  mCHP (6.6%)  mBP (100.0%) | mINP (0%)  mIDP (0%)  mOP (0%) |
| Bisphenols | Bisphenols compounds are also the parent | Bisphenol A (48.2%)  Bisphenol F (24.5%)  Bisphenol S (100%) | Bisphenol AF (0%)  Bisphenol AP (0%)  Bisphenol B (0%)  Bisphenol P (0.7%)  Bisphenol Z (0%) |
| Other Compounds | Phthalic acid  DINCH | Phthalic acid (13.2%)  mCOCH (78.0%)  mONCH (46.1%)  mHNCH (70.4%) |  |

**Caption:**

LMW-P parent compounds – DMP: di-methyl phthalate; DEP: di-ethyl phthalate; DiBP: di-isobutyl phthalate; DBP: di-butyl phthalate; DnHP: di-n-hexyl phthalate; DHPP: di-2-heptyl phthalate.

LMW-P metabolites – mMP: mono-methyl phthalate; mEP: mono-ethyl phthalate; mIBP: mono-isobutyl phthalate; mBP: mono-butyl phthalate; mHxP: mono-hexyl phthalate; mHpP: mono-heptyl phthalate.

HMW-P parent compounds – DEHP: di-2-ethylhexyl phthalate; DiNP: di-isononyl phthalate; DiDP: di-isodecyl phthalate; DnOP: di-n-octyl phthalate; BzBP: benzylbutyl phthalate; DCHP: di-cyclohexyl phthalate; DPHP: di-(2-propylheptyl) phthalate

HMW-P metabolites – mECPP: mono-(2-ethyl-5-carboxypentyl) phthalate; mCMHP: mono-(2-carboxymethyl-hexyl) phthalate; mEHHP: mono-(2-ethyl-5-hydroxyhexyl) phthalate; mEOHP: mono-(2-ethyl-5-oxohexyl) phthalate; mEHP: mono-2-ethylhexyl phthalate; mINP: mono-isononyl phthalate; mCIOP: mono-(carboxyisooctyl) phthalate; mCINP: mono-(carboxyisononyl) phthalate; mIDP: mono-isodecyl phthalate; mCPP: mono-(3-carboxypropyl) phthalate; mCHpP: mono-carboxy-heptyl phthalate; mOP: mono-octyl phthalate; mBzBP: mono-benzyl phthalate; mCHP: mono-cyclohexyl phthalate; OH-mPHP: mono-(2-propyl-6-hydroxyheptyl) phthalate; oxo-mPHP: mono-(2-propyl-6-oxoheptyl) phthalate; cx-mPHxP: mono-(2-propyl-6-carboxyhexyl) phthalate

Other Compounds – DINCH: 1,2-cyclohexane dicarboxylic acid diisononyl ester; mCOCH: cyclohexane-1,2-dicarboxylic acid mono carboxyisooctyl ester; mONCH: cyclohexane-1,2-dicarboxylic acid mono(oxo-isononyl) ester; mHNCH: cyclohexane-1,2-dicarboxylic acid mono(hydroxy-isononyl) ester

**SUPPLEMENTAL FIGURE 1: Comparisons of Phthalate Levels to Historical NHANES Data**


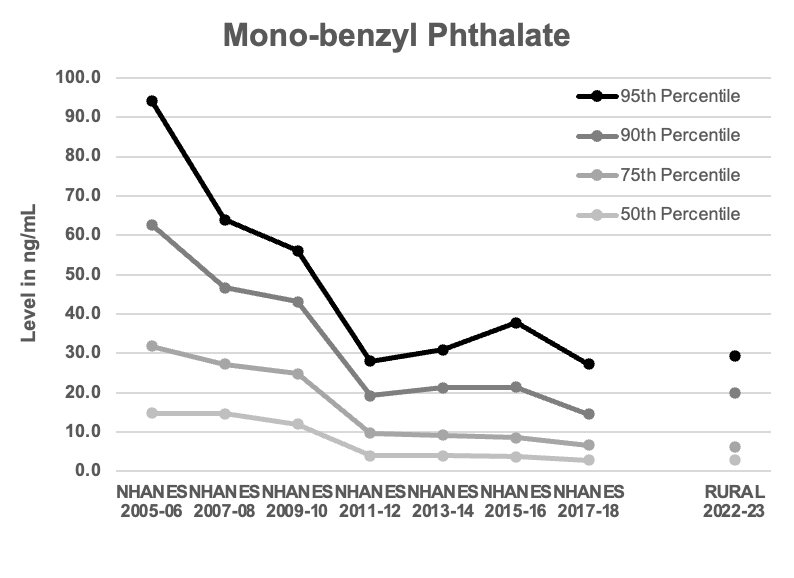

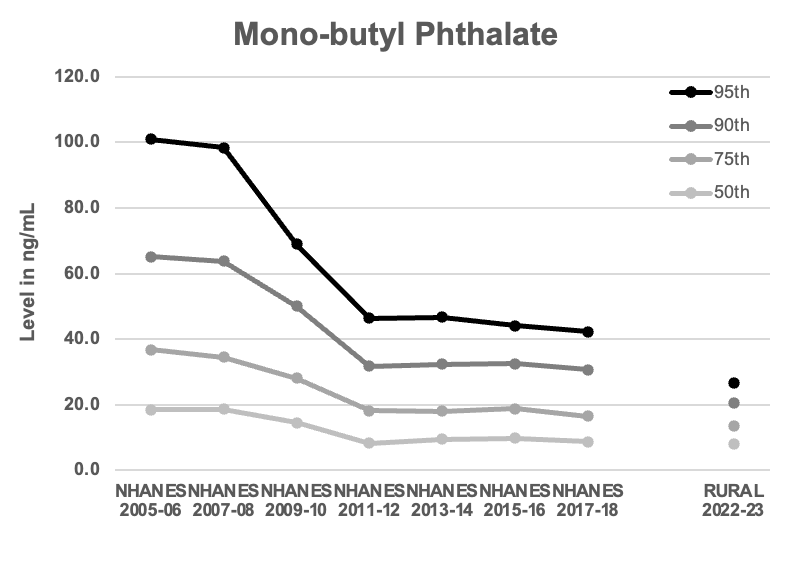


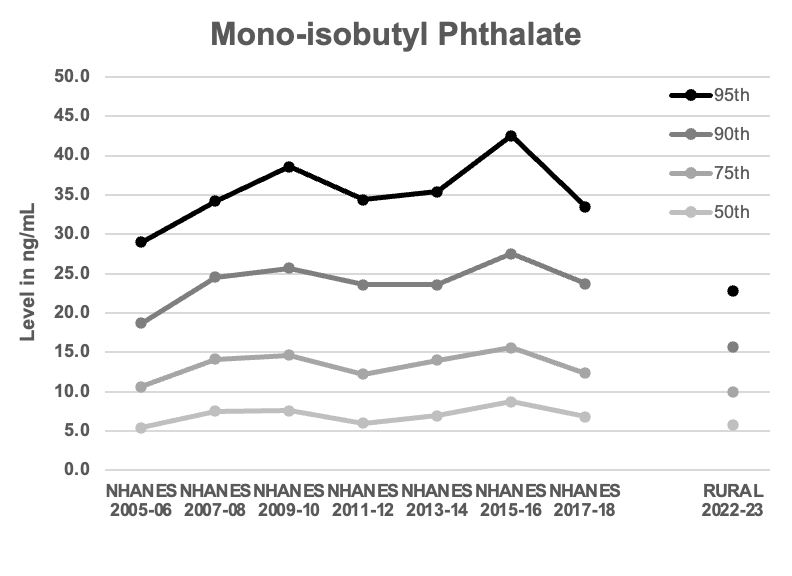

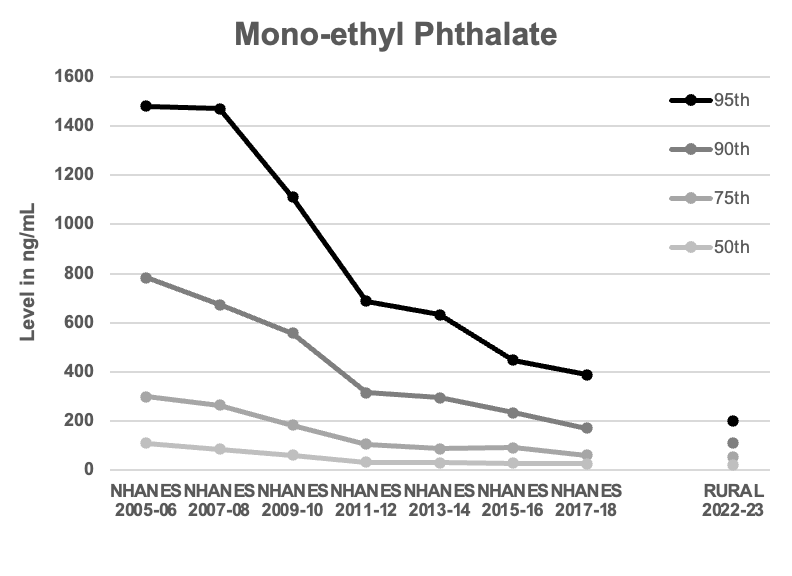


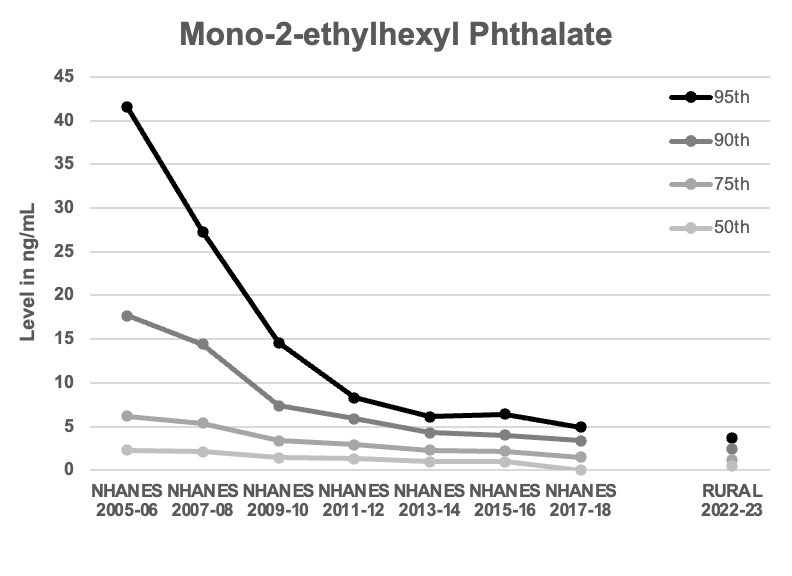

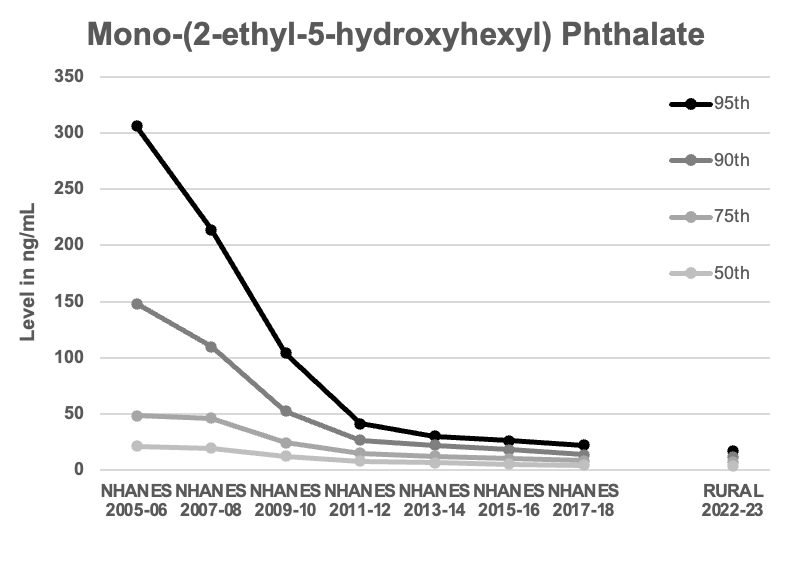


**SUPPLEMENTAL FIGURE 2: Additional Comparisons of Phthalate Levels to Historical NHANES Data**


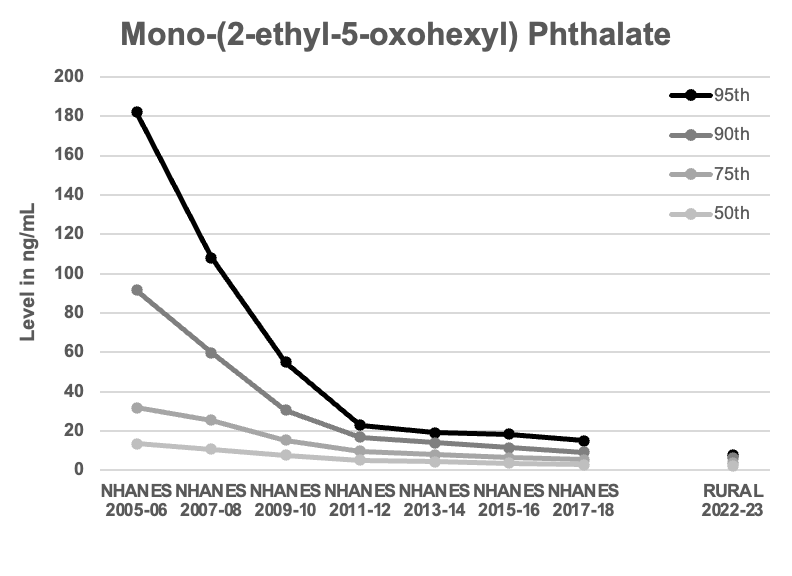

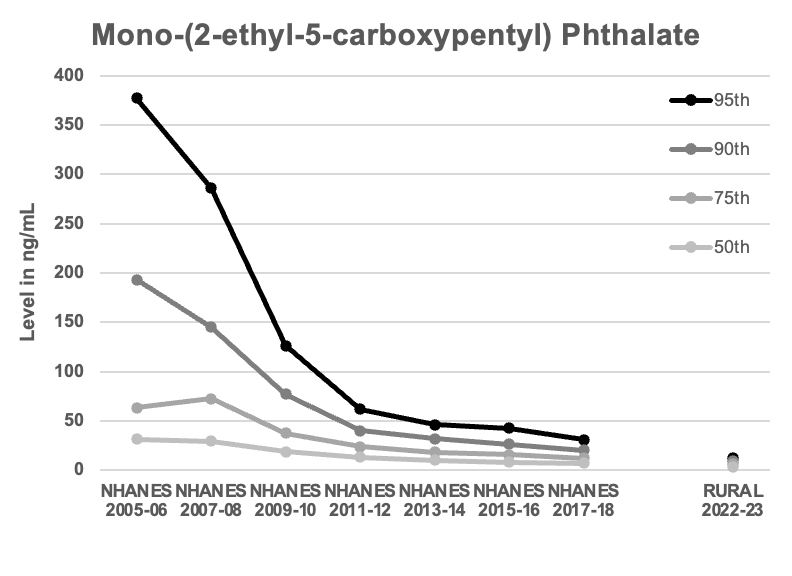


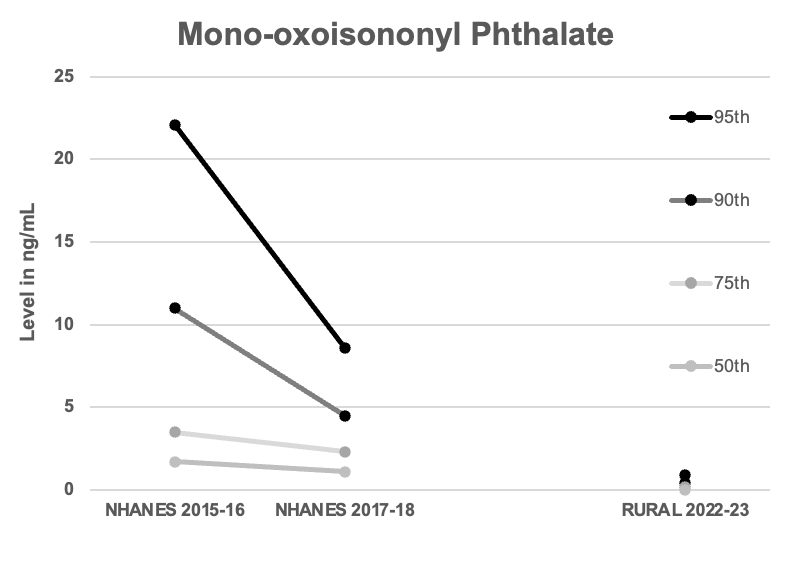

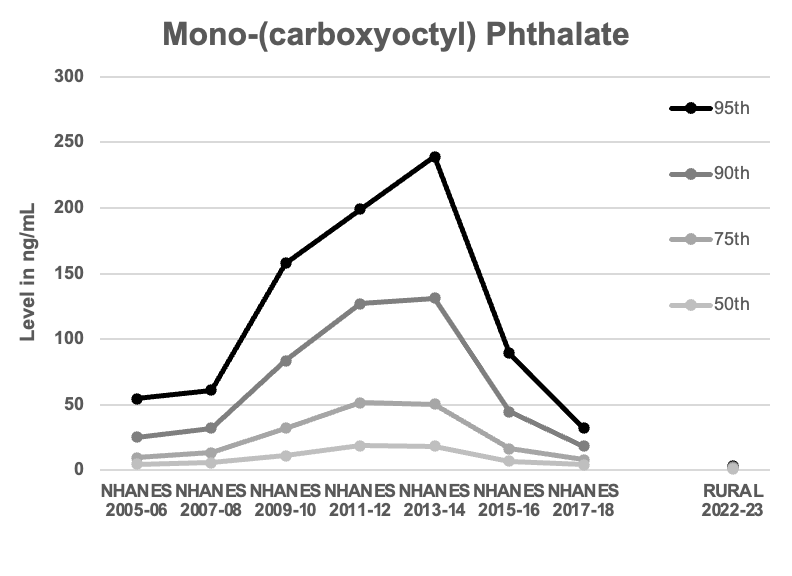


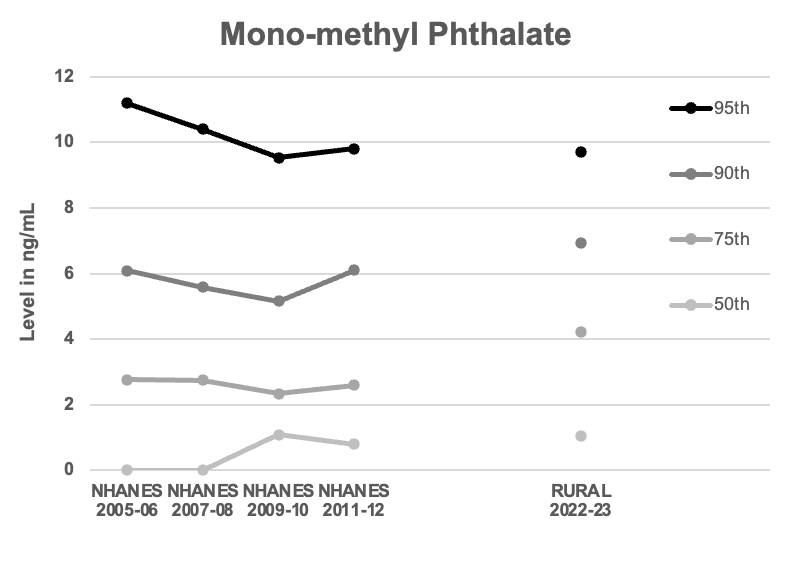

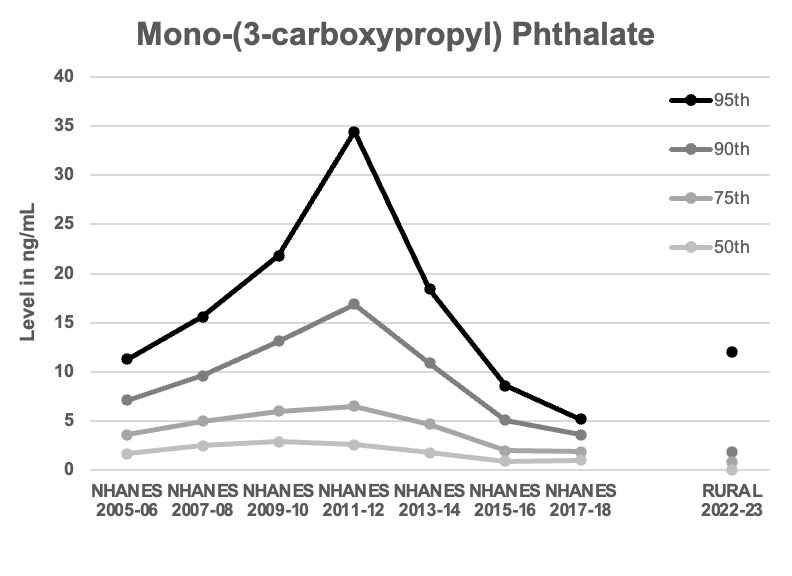


**SUPPLEMENTAL FIGURE 3: Comparisons of Non-Phthalate Alternative Levels to Historical NHANES Data**


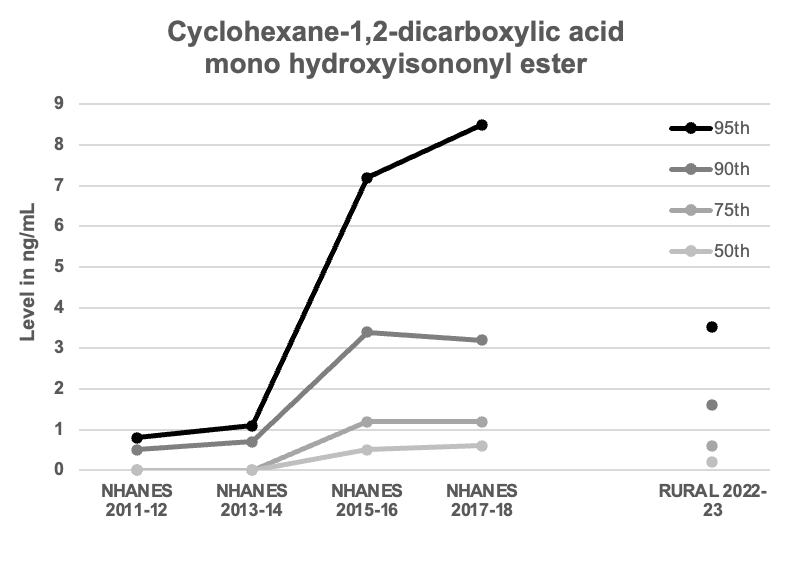

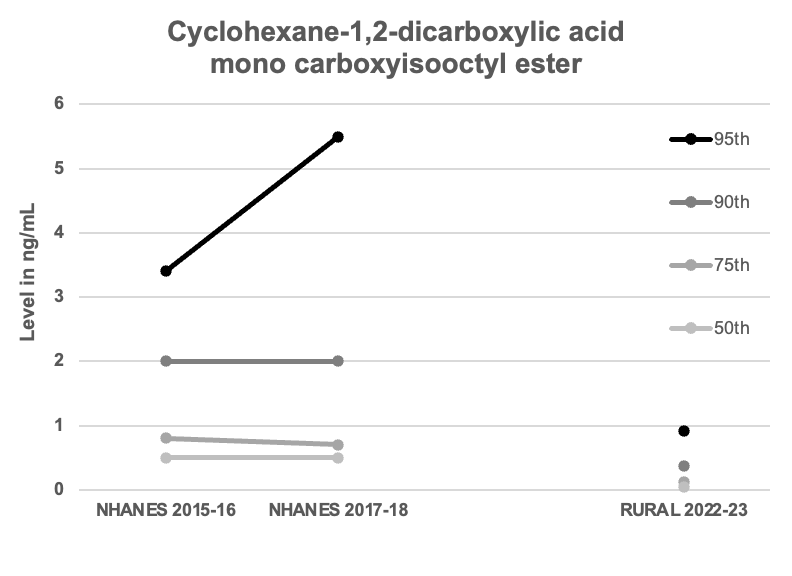

Supplement: Supplementary file 1 — Supporting Information S1 [file DMRR-42-e70177-s001.docx]
